# Bubbles determine the amount of alcohol in Mezcal

**DOI:** 10.1038/s41598-020-67286-x

**Published:** 2020-07-03

**Authors:** G. Rage, O. Atasi, M. M. Wilhelmus, J. F. Hernández-Sánchez, B. Haut, B. Scheid, D. Legendre, R. Zenit

**Affiliations:** 1 Instituto de Investigaciones en Materiales, Universidad Nacional Autónoma de México, Apdo. Postal 70-360, Coyoacan, Ciudad de Mexico, 04510 Mexico; 2 TIPs (Transfers, Interfaces and Processes), Université Libre de Bruxelles, Avenue F.D, Roosevelt, 50, CP 165/67, 1050 Brussels, Belgium; 3 Institut de Mécanique des Fluides de Toulouse, Université de Toulouse, CNRS, Toulouse, France; 4 Soft Matter, Fluidics and Interfaces, MESA+ Institute for Nanotechnology, University of Twente, P.O. Box 217, 7500 AE Enschede, The Netherlands; 5 Department of Mechanical Engineering, University of California, Riverside, 3401 Watkins Drive, Bourns Hall A313, Riverside, CA 92521 USA; 6 Instituto de Ciencias Aplicadas y Tecnología, Universidad Nacional Autónoma de México, Circuito Exterior s/n, Ciudad de México, 04510 Mexico; 7 Center for Fluid Mechanics, School of Engineering, Brown University, 184 Hope St, Providence, RI 02912 USA

**Keywords:** Chemical engineering, Mechanical engineering, Fluid dynamics

## Abstract

Mezcal is a traditional Mexican spirit, obtained from the distillation of fermented agave juices. Its preparation has been conducted for centuries in an artisanal manner. The method used to determine the correct alcohol content is of particular interest: a stream of the liquor is poured into a small vessel to induce surface bubbles. These bubbles, known as pearls by the Mezcal artisans, remain stable for tenths of seconds only if the alcohol content is close to 50%. For higher or lower alcohol content, the bubbles burst rapidly. The long bubble lifetime is the result of surfactant-induced surface tension changes. However, the precise mechanism and its relation to alcohol content remain unexplained. In this investigation, the extended lifetime of pearls was studied both experimentally and numerically. It was found that changes in surface tension, density, viscosity (resulting from mixing ethanol and water), and the presence of surfactants are all relevant to extend the bubble lifetime. The dimensionless bubble lifetime was found to reach its maximum value when the Bond number was close to unity, corresponding to 2 mm Mezcal bubbles. These findings show that the traditional empirical method does work. Beyond this, the understanding of the process provides physical insight to many other natural and industrial problems for which the stability of surface bubbles is of importance, such as bio-foams, froth floatation, and volcanic flows.

## Introduction

The consumption of alcoholic drinks can be traced back to the appearance of early humans. While its inception may have resulted from the need to find reliable sources of potable liquids^1^, these drinks have become part of the cultural identity of many societies^2^. Most cultures around the world are known for a ‘local’ drink. In the case of Mexico, the most popular distilled spirit is Tequila which, belongs to a wider class of distilled agave-based products that are similar in production^3^ but vary regionally. It was believed that the production of agave-distilled spirits began with the arrival of Europeans by the end of the 16th century^4^. However, recent findings indicate that alcohol distillation was known in Mesoamerica long before, for at least 25 centuries^5^. Our study focuses on Mezcal, which has progressively gained worldwide recognition and has distinctive cultural value in rural villages in Oaxaca^6^. Although its production and denomination are normed^7^, its preparation remains mostly artisanal^8^.

**Figure 1:**
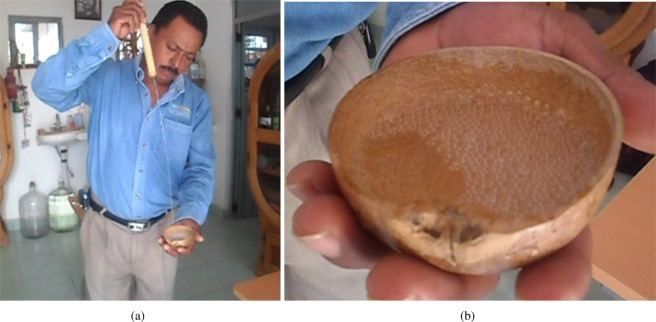
(**a**) Traditional technique to form superficial bubbles (pearls); see also the Video S1 in the Supplementary Information section. (**b**) Zoom of the pearls of Mezcal in a gourd cup (*jícara*). Images taken by L. Diaz-Damacillo, reproduced with permission.

According to popular accounts (informally documented by interviews with artisans) and a few formal reports^9^, the traditional method employed to determine the alcohol content in Mezcal consists of inspecting the lifetime of bubbles that are formed by splashing a jet of the liquor into a small container (Fig. 1(a) and S1 Video in the Supplementary Information section). As a result of the continuous splashing, the liquid surface breaks and so-called pearls form. If the amount of alcohol in the liquid is correct (about 55% volume fraction of ethanol), pearls persist for up to tenths of seconds, see Fig. 1(b). The method is surprisingly accurate.

Interestingly, a similar technique has also been used to determine the alcoholic content in other spirits. Davidson^10^, for example, conducted experiments on the foam stability of Bourbon diluted with different amounts of water. The technique is essentially the same as that presented here and shows the same phenomenon: for volumetric contents of alcohol of about 50%, the superficial bubbles are notably more durable than in other mixtures. Ahmed and Dickinson^11^ conducted experiments for whiskey and found similar results. They argue that the changes in bubble lifetime are related to the changes in solubility of surfactant molecules present in this type of beverage. In contrast, Tuinier *et al*.^12^ found an extended foam-life for ethanol volume fractions close to 10%. In none of the previous studies, the precise mechanism responsible for the extended bubble life duration has been explained.

## Lifetime of surface bubbles

Due to its relevance to many processes beyond the production of water-alcohol mixtures, e.g. bubbles on the surface of the ocean^13,14^, fish nests^15^, froth flotation^16^ and vulcanology^17^, the time of residence of a superficial bubble on a free surface has been extensively studied. How long a bubble remains floating on a surface depends on the drainage of the film between the bubble and the surface, which results from the balance between two competing effects (gravitational and capillary induced drainage) and viscous forces. The film reduces its thickness in time, and when it is sufficiently thin, it spontaneously pierces and breaks. Note that the precise mechanism that determines the film rupture thickness is not known^18^.

Two additional factors affect bubble lifetime during Mezcal testing: the alcohol content and the presence of surfactants. Ethanol and water are the main components in Mezcal, with volume fractions of the former ranging from 36 to 55%, according to the norm^7^. The effect of alcohol on bubble lifetime is not fully understood yet as evaporation can either shorten the lifetime of films^19^ or extend it^20^. Indeed, some studies suggest that alcohol can increase bubble stability^11^, while others have shown it to have a destabilizing effect^21^. While the volatility of ethanol may induce Marangoni flows^19^, the properties of water-ethanol mixtures show a non-monotonic behavior with ethanol content^22^, making it difficult to understand the physical mechanisms behind film drainage. Furthermore, it has been long recognized that surfactants significantly alter the drainage process of bubble films^23^, thereby affecting the lifetime. In addition, Mezcal has many other compounds (in small fractions) that can act as surfactants^24^, such as methanol, acetic acid, ethyl acetate, higher alcohols, esters, ketones, furanes, acetals, aldehydes, phenols, and terpenes as well as proteins, which can have a significant effect in delaying the drainage of bubble films as in the case of beer^25^.

### Physical mechanisms that produce surface tension gradients

Three physical mechanisms may induce gradients of surface tension: surfactant transport, heterogenous evaporation, and local changes in temperature. All of these mechanisms affect the time the film takes to drain and, therefore, bubble lifetime. Let us first consider the effect of alcohol concentration. The addition of 10% of mass of ethanol to water, for example, decreases the surface tension of the mixture more than 30% as seen in the study by Vazquez *et al*.^26^, where variations in temperature were shown to have a small effect —an increase of 30 C resulted in a surface tension reduction of less than 6%.

In ethanol-water mixtures, the alcohol evaporates at a constant rate regardless of the initial concentration^27^. Therefore, it would be expected for the evaporation rate to be uniform along the film, avoiding gradients of surface tension. However, the evaporation rate does vary with the thickness of the film as in Mezcal pearls. Such non-uniform evaporation naturally results in a decrease of bubble lifetime. Hence, we do not discard the effect of evaporation. However, given the long lifetimes observed experimentally, we consider this effect as secondary. We can thus argue that surface tension gradients result primarily from the changes in the surfactant concentration. To fully resolve this issue, a numerical analysis would have to account for both evaporation and thermal effects. Such a scheme is beyond the scope of the present work. Instead, based on the arguments above, we account only for the surfactant-induced Marangoni effect. The details are explained in the following sections.

The aim of our investigation is to demonstrate that the traditional technique to assess the ethanol content in Mezcal actually works and to explain the relationship between the lifetime of pearls and alcohol content. Although modern methods of determining alcohol content are accessible and reliable, even for small remote rural communities, the fundamental understanding of the physical mechanisms behind bubble and foam stability is relevant due to their importance to many other fields. In this study, we conducted experiments to determine the lifetime of surface bubbles of many different types of Mezcal and water-ethanol mixtures. To gain insight into the film drainage process, we also conducted numerical simulations and analyzed all results in terms of dimensionless quantities.

### Dimensional analysis

As proposed by Barenblatt^28^, dimensional analysis can be used to understand a physical phenomenon more deeply. There are several ways in which the relevant dimensionless numbers can be identified. We consider two alternatives below.

### Dimensionless groups by simple inspection

Drainage results from a balance between viscous forces and either gravitational or surface tension forces. The ratio that determines the relative importance between gravitational and surface tension effects is the Bond number,

1 $$Bo=\frac{\rho g{D}^{2}}{\sigma },$$

where *ρ* is the liquid density, g is the gravitational acceleration, *D* is the bubble diameter, and *σ* is the surface tension. All of these quantities are attainable via laboratory experiments. Here, *D* is determined by analyzing images of bubbles from a top view (Methods Section). It should be noted that this metric gives an apparent bubble size rather than an equivalent bubble diameter, which measures the diameter of a spherical bubble with equivalent volume.

Considering surface tension effects, the bubble lifetime can be scaled by incorporating viscous effects leading to:

2 $${T}_{life}^{\ast }=\frac{{T}_{life}\sigma }{\mu D},$$

where *μ* is the liquid viscosity. One can propose the following functional relationship between ${T}_{life}^{\ast }$ and *Bo*:

3 $${T}_{life}^{\ast }=\Phi (Bo).$$

Now, as explained above, the ever-presence of surfactants plays an important role in the film drainage process. If one defines Δσ as the change in surface tension resulting from surfactant gradients along the interface, and the relative variation as

4 $$\Pi =\frac{\Delta \sigma }{\sigma },$$

one can therefore argue that

5 $${T}_{life}^{\ast }=\Phi {\prime} (Bo,\Pi ).$$

Here, we aim to understand the effect of each group by considering that

6 $${T}_{life}^{\ast }=\Phi (Bo)\cdot \Psi (\Pi ).$$

In other words, we shall consider that the effect of surfactants can be de-correlated from the drainage. This fact is not obvious and represents an important assumption of our study. However, as discussed below, the assumption appears to be reasonable. Furthermore, the analysis indicates that the surfactant-induced (Marangoni) stresses should be considered to determine an appropriate timescale.

### Dimensionless groups from the analysis of the flow type

The paper by Champougny *et al*.^23^ discusses the unsolved problem of bubble drainage in the presence of surfactants. They propose an ad-hoc model based on an extrapolation length *λ* that allows describing the transition from stress-free interfaces (*λ* = ∞) to no-slip interfaces (*λ* = 0). The flow between stress-free interfaces is a plug flow at leading-order, dominated by extensional viscous stresses. Debregeas *et al*.^29^ have shown that the film thickness of such a ‘bare’ bubble decays exponentially in time and that the film always ruptures at the apex. Contrarily, the flow between no-slip (rigid) interfaces is a Poiseuille-like flow dominated by viscous shear stresses (as described, for instance, in^30^). Now, surfactant-induced Marangoni stresses have been shown to rigidify, at least partially, the interfaces, such that the flow is essentially a shear flow (e.g., Champougny *et al*.^31^ for film formation or Atasi *et al*.^32^ for confined bubbles in microchannels, both in the presence of surfactants). Even traces of surfactants, such as impurities, have been found to induce dominant Marangoni stresses^33^. Additionally, local thinning mechanisms of the film have been observed in soap bubbles^18^, reminiscent to marginal regeneration^34^, and eventually leading to the rupture of the film away from the apex. Based on these arguments, we conjecture that viscous shear stresses dominate the film drainage. This hypothesis is corroborated below via numerical simulations.

In absence of a simple model available that accounts for the presence of surfactants and with the aim to find an appropriate timescale, let us consider a drainage flow dominated by viscous shear, balanced either by gravity or capillarity. Starting with a gravity-driven drainage, the momentum balance at leading order in the lubrication approximation writes

7 $$\mu {\partial }_{yy}u=\rho g,$$

where the cross-stream coordinate *y* in the film can be scaled with the critical thickness for rupture *h*_*rup*_. The choice of this length scale is justified by the fact that the drainage dynamics is not significantly influenced by the initial condition since the main contribution to the lifetime occurs at the later stage, i.e. when the film is the thinnest, which is also when the viscous dissipation dominates. Note, however, that *h*_*rup*_ is not a quantity that can be obtained directly from the experiments. By considering the speed at which the film retracts after it ruptures, as explained in the Methods Section below, an estimate of this thickness can be inferred. For simplicity, we consider that the rupture always occurs at the same thickness value. Considering that *u* ~ *D/t*_*g*_, the timescale for drainage driven by gravity can be obtained as:

8 $${t}_{g}=\frac{\mu D}{\rho g{h}_{rup}^{2}}\mathrm{}.$$

Similarly, the momentum balance for a capillary-driven drainage is given by

9 $$\mu {\partial }_{yy}u={\partial }_{x}P,$$

where the streamwise coordinate *x* can be scaled with *D*. If we assume that the pressure *P* results from a Laplace effect, we can write *P* ~ *σ*/*D*. By estimating that in this case *u* ~ *D/t*_*c*_, the timescale for drainage driven by capillarity is given by

10 $${t}_{c}=\frac{\mu {D}^{3}}{\sigma {h}_{rup}^{2}}.$$

Comparing these two timescales leads to the Bond number, namely $Bo={t}_{c}/{t}_{g}$, exactly as defined in Eq. (1).

It is interesting to note that the Bond number plays two roles in this problem, a static and a dynamic one. On the one hand, it allows evaluating the static shape of the interfaces, namely a spherical bubble underneath an almost undeformed liquid surface for $Bo\ll 1$, and a deformed bubble under a deformed liquid surface for $Bo\gg 1$. On the other hand, as explained above, it provides a way to evaluate the dominant driving force for drainage, namely the capillary force for $Bo\ll 1$ and the gravity force for $Bo\gg 1$. The striking feature of the present problem is that $Bo\sim 1$ indicates a transition which, as shown below, coincides with the maximum dimensionless bubble lifetime.

A different dimensionless lifetime can, therefore, be defined in terms of the capillary timescale, *t*_*c*_:

11 $${T}_{life}^{\ast \ast }=\frac{{h}_{rup}^{2}}{{D}^{2}}\frac{{T}_{life}\sigma }{\mu D}={\varepsilon }^{2}{T}_{life}^{\ast }$$

where ${T}_{life}^{\ast }$ is defined in Eq. (2). We also introduce $\varepsilon ={h}_{rup}/D\ll 1$, a measure of the slenderness of the film. Equation (11) indicates that the viscous forces act perpendicularly to the capillary/gravity forces, as reminiscent to shear-dominated flow. Conversely, forces in the case of an extensional flow would act along in the flow direction affecting the drainage; for such a case *ε* would be close to unity. Consequently, ${T}_{life}^{\ast }$ would be the appropriate group for an extensional drainage. In the present problem, ${\varepsilon }^{2} \sim {10}^{-4}$, therefore ${T}_{life}^{\ast \ast }$ is more appropriate in the present context.

As will be shown later, the data shows a clear transition in trend at around *Bo* ~ 1. Using the ${T}_{life}^{\ast \ast }$ scale, we can derive the trends in both limits of large and small Bond numbers. In the limit of large Bond number the drainage is governed by gravity, therefore, using Eq. (8), one can write

12 $${T}_{life}^{\ast \ast }\propto B{o}^{-1}\,(Bo\ll 1).$$

On the other hand, when the Bond number is small the drainage is dominated by capillarity, which, considering Eq. (10), implies that

13 $${T}_{life}^{\ast \ast }\propto 1\,(Bo\ll 1).$$

These two trends will be contrasted against the experimental results below.

### Review of relevant models to predict the bubble lifetime

As stated in the Introduction, many studies have addressed the issue of bubble lifetime. Here, we summarize some relevant studies. Note that we do not aim to comprehensively summarize the state-of-the-art of the subject. To draw comparisons with our experiments and among the different models, we recast the predictions in terms of the dimensionless groups discussed above and recognize that for a given liquid, varying the Bond number amounts to varying the bubble size.

#### Large bubbles

*Bo* ≫ 1

In the limit of large Bond numbers, the bubbles are large and the film near the apex is essentially uniform and the drainage is driven by gravity.

For the case of rigid interfaces, an analytical solution exists for the dimensionless lifetime as follows^35^

14 $${T}_{life}^{\ast \ast }=\frac{3}{2}B{o}^{-1}\mathrm{}.$$

Notably, this prediction underestimates most of the experimental times shown later in the Results section, which indicates that the Marangoni stress in the experiments is large enough to retard the drainage longer than what rigid interfaces would do. This is only possible if the mean flow is upwards, i.e. towards the apex, entrained by Marangoni stresses. Such gradient-tension-induced stresses would need to built up prior to the drainage phase^36^. The simulations shown below show this type of behavior.

In the case of stress-free interfaces, Kocarkova *et al*.^37^ calculated the evolution of the film thickness considering an extensional flow in the film, assumed to be uniform and axisymmetric. They observed an exponential thinning, from which the dimensionless lifetime can be shown to be:

15 $${T}_{life}^{\ast } \sim B{o}^{-1}\mathrm{}.$$

Champougny *et al*.^23^ extended the analysis of Kocarcova *et al.*^37^ considering certain levels of surface rigidification of the interfaces to account for the presence of surfactants. They also found an exponentially thinning of the film with time, which leads to the same functional relation of Eq. (15) but the proportionality constant was larger in the case of surfactants. Interestingly, they also found that the puncture of the bubble film changed from the apex to the foot of the bubble as the amount of surfactants increased.

Finally, Lhuissier and Villermaux^18^ considered the case of bubbles in water. They argued that the scaling of a viscous drainage was not appropriate for water due to the phenomenon known as marginal regeneration^38^. In short, the balance of capillary pressure from the film curvature, the meniscus at the foot of the bubble and surface tension gradients lead to a non-uniform film thickness which causes the appearance of a localized pinching. Moreover, Lhuissier and Villermaux^18^ recognized that the film breakup could also be influenced by the Bénard-Marangoni convection flows within the film. Considering the fluctuations from marginal regeneration convection cells and probabilistic arguments of the puncture breakup mechanism, they found

16 $${T}_{life}^{\ast } \sim B{o}^{-1/4},$$

which also indicates a decreasing lifetime with an increase of *Bo*, as qualitatively obtained in Eq. (12).

#### Small bubbles

*Bo* ≪ 1

In the limit of small Bond numbers, no simple model exists for the bubble lifetime because in this limit the drainage is driven by capillary forces which also induce surface deformations. The model of Howell^39^, considers this case but assumes an extensional flow, which leads to

17 $${T}_{life}^{\ast }={\kappa }_{1}B{o}^{\mathrm{1/2}}\,(Bo\ll \mathrm{1),}$$

where *k*_1_ is a constant that depends on the initial and rupture thicknesses. This expression indicates that the rupture time increases with Bo: the larger the bubble, the longer it will take to burst. This calculation assumes that the film drains uniformly and axisymmetrically without surfactants. The small *Bo* limit indicates that the bubble is nearly spherical and is mostly immersed below the liquid free surface. Note that this trend is different from that obtained considering a shear flow (Eq. 13).

#### Transition-sized bubbles

*Bo* ~ 1

From the brief review shown above, an important conclusion can be reached: the trends are opposite for small and large Bond numbers. This fact indicates that there is a transition at a certain value of *Bo*. From our arguments, confirmed below by the experiments, the critical *Bo*-value is around unity, where capillary and gravity forces are of comparable magnitude to drive the drainage. And this transition corresponds to a maximum dimensionless lifetime, as suggested by the cross-over between the models given above.

## Experimental Results

### Pearl formation

**Figure 2:**
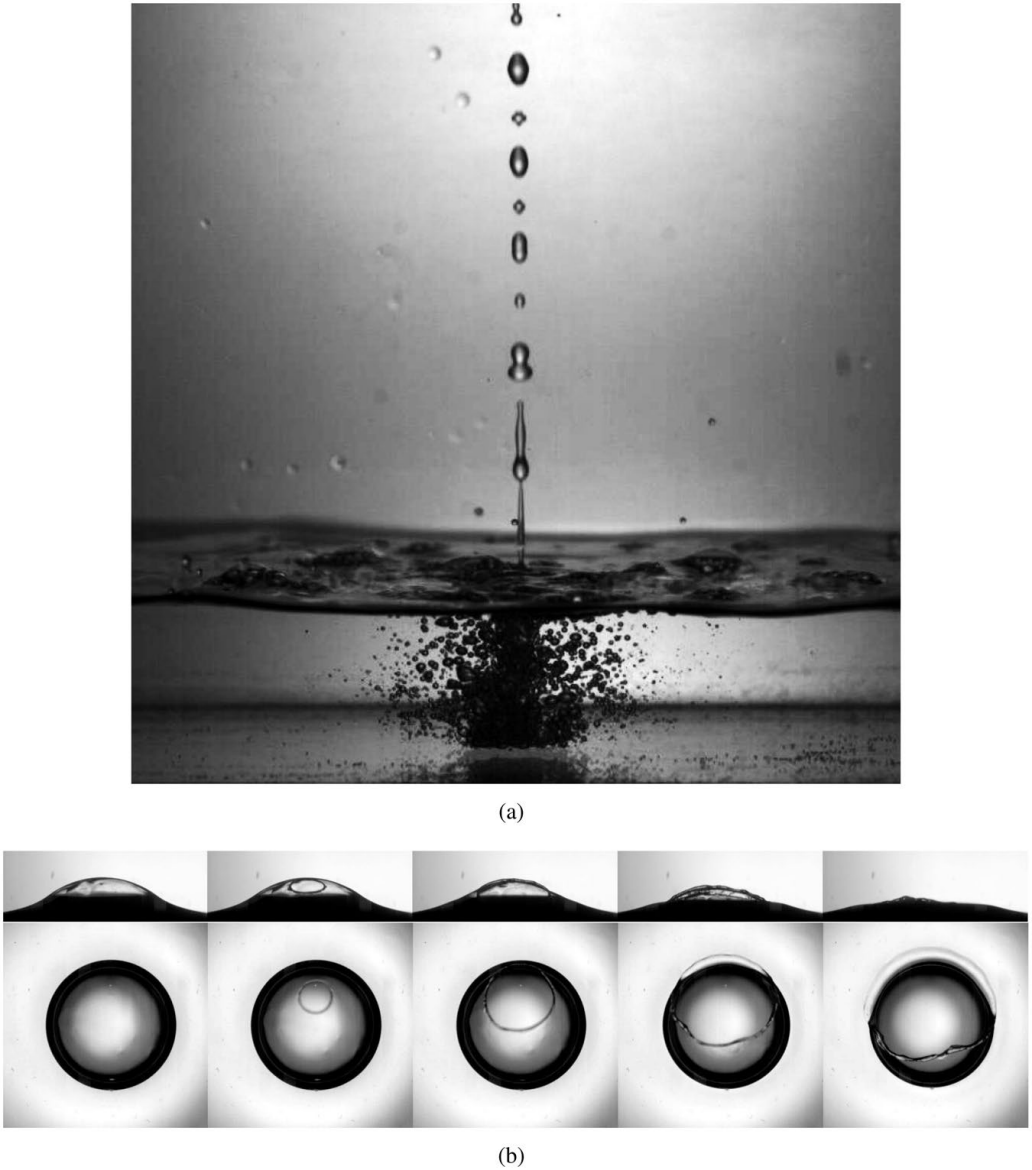
(**a**) Reproduction of the formation process in the laboratory. (**b**) The bursting of a surface bubble. The images were taken from the side and top, simultaneously. The time between frames is 0.4 ms. The diameter of the bubble is 1.9 mm, corresponding to *Bo* = 1.06. The liquid is Mezcal M1, from Table 1.

Figure 2(a) shows an image of the formation process of pearls (see also Video S2, Supplementary Information section), considering the controlled experiment described in the Methods Section. The fluid jet fragments into droplets, which continuously splash against the surface of the liquid. The continuous splashing induces the formation of air cavities that, in turn, form bubbles that eventually rise to the surface. However, only bubbles of a certain size (of approximately 2 mm in diameter) remain on the surface for longer times.

The process of air entrainment from a plunging jet has been studied in detail^40–42^, due to its relevance for many flow phenomena such as aeration of the ocean and other water reservoirs. It is believed to be well understood. Hence, we did not pursue a more complete study of the formation process. Instead, we focus on the residence time of bubbles on the surface.

### Bubble lifetime

Figure 2(b) shows a time sequence of a typical Mezcal bubble at the moment of bursting (see also Video S3, Supplementary Information section), produced with the system described in the Methods Section. It was observed that bursting initiates with a small hole rupturing the film, which opens quickly until it retracts completely. The puncture does not typically appear at the apex of the bubble; the thinning of the film is not uniform arguably due to the partial regeneration mechanism^18^.

Several experiments were conducted under the same nominal conditions (same fluid and same bubble diameter) to calculate the typical value of the rupture thickness, according to the scheme described in the Methods Section. It was found that the thickness was *h*_*rup*_ = 23.5 ± 4.8 *μ*m for the image shown in Fig. 2(b). This value is relatively large, compared to what has been measured for the case of water and seawater^18^. This value was used as a reference for the numerical simulations and is also discussed in the scaling of the bubble lifetime.

**Figure 3:**
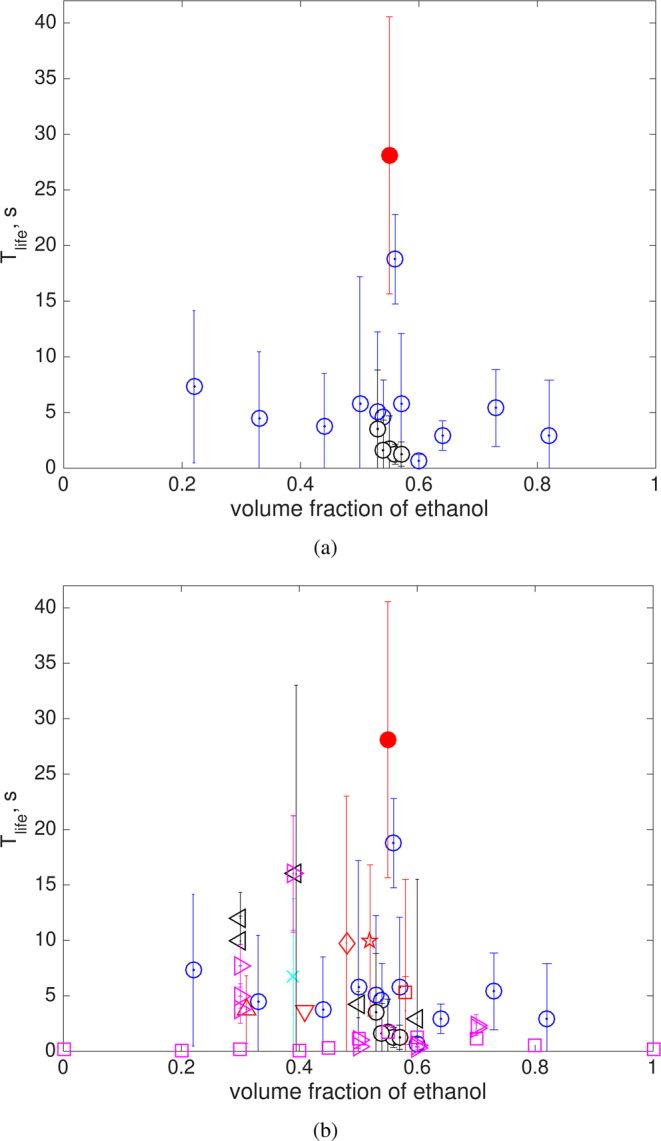
(**a**) Bubble lifetime as a function of ethanol concentration for Mezcal (M1, ); Mezcal with the addition of water or ethanol (MA1, ); Mezcal with the addition of 55% water-ethanol mixture (MA2, ○). (**b**) Bubble lifetime as a function of ethanol concentration for all tested liquids. Symbols as in Table 1.

Experiments were initially conducted using Mezcal with the ‘correct’ ethanol volume fraction, identified as M1 (see Table 1). The mean lifetime in this case was 28.1 ± 12.5 s, depicted in Fig. 3(a) by the filled red circle. To vary the amount of ethanol in this liquid, either pure water or ethanol was added. The measured lifetimes for this ethanol-adjusted Mezcal are shown Fig. 3(a), denoted as liquid MA1 from Table 1. The figure shows the measured bubble lifetime as a function of ethanol volume fraction. As the amount of ethanol increases or decreases, the bubble lifetime decreases. A clear sharp maximum was observed for the Mezcal sample with 55% ethanol. It should be noted that even though the amount of other components was diluted (by adding water or ethanol), increasing or decreasing the alcohol content led to a measurable variation of the lifetime. Therefore, based on these observations, we conclude that the traditional technique does work: the bubble lifetime shows a maximum value for a certain intermediate value of ethanol in Mezcal. Although the experiments were conducted in a controlled environment (standard laboratory conditions), a large variability of the measured lifetimes was found. Large scatter is often reported in experimental reports of the lifetime of bubbles, especially with water and other surface-contaminated liquids^13,18^. To our knowledge single bubble lifetime measurements for water-alcohol mixtures have not been reported to date, but similarly large scatter can be expected due to evaporation effects^19^. In the present case, the large uncertainty bars in our measurements may also result from the use of unfiltered artisanal Mezcal. According to the norm^7^, particulate matter is expected to be present. The presence of particles, and maybe fibers, would also explain the relatively large bursting thickness of the film.

**Table 1:** Physical properties of liquids used in the investigation. M: Mezcal, MA: modified Mezcal, W: water, E: ethanol.

Fluid	type or brand agave species	density *ρ*, kg/m^3^	viscosity *μ*, mPa s	surface tension *σ*, mN/m	ethanol content % vol
M1 ()	Los Sánchez espadín	919.0	3.07	30.6	55
MA1 ()	M1 + W or E espadín	906.2 to 965.8	1.83 to 3.03	25.0 to 41.0	22 to 82
MA2 ()	M1 + W/E (55/45) espadín	897.4 to 901.2	2.82 to 2.89	28.4 to 29.1	53 to 57
M2 ()	El Amate espadín	914.4	2.98	28.7	48
M3a ()	Los peregrinos (cuerpo) espadín	912.6	2.66	29.2	41
M3b ()	Los peregrinos (punta) espadín	926.9	3.07	29.2	58
M3c ()	Los peregrinos (cola) espadín	933.8	2.34	32.7	31
M4 ()	Los peregrinos (MC) Madre Cuishe	928.3	3.08	29.9	52
M5 ()	Los peregrinos (reposado) espadín	949.1	2.58	34.0	39
M6 ()	Real Minero largo	850.0 to 950.0	2.30 to 3.06	25.9 to 31.6	30 to 70
M7 ()	Matatlan tobalá	850.0 to 950.0	2.30 to 3.06	25.8 to 32.0	30 to 70
WE ()	W-E mixtures N/A	791.0 to 1000.0	1.10 to 3.01	23.0 to 72.0	0 to 100

To evaluate the effect of surfactants and other components, a second set of experiments was conducted. In this set, different amounts of a 55% water-ethanol mixture were added to the sample M1, also shown in Fig. 3(a) and denoted as liquid MA2 in Table 1. In this manner, the amount of ethanol remains approximately constant but the quantity of surfactants is diluted with respect to the original sample. The result is clear: the pearl lifetime is significantly reduced compared to the original sample, even though the volumetric concentration of ethanol is nearly the same. We can, therefore, argue that the amount of surfactants is an important factor to determine the lifetime of pearls.

**Figure 4:**
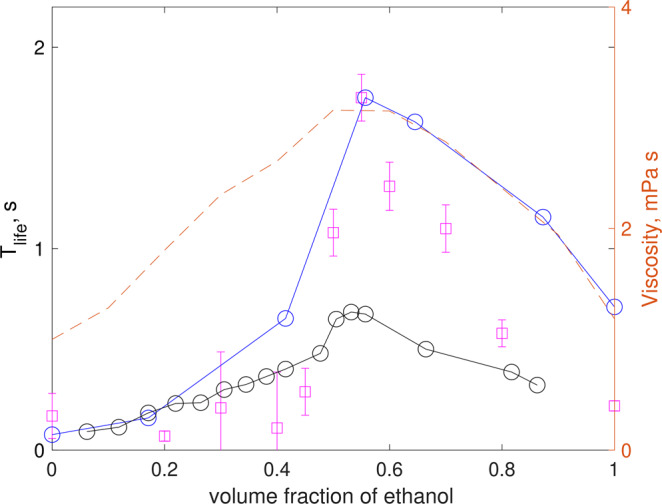
Bubble lifetime as a function of ethanol concentration for water/ethanol mixtures: experiments (, *D* = 2.77 ± 0.83 mm), error bars denote the standard deviation of the measurements; numerical results (, *D* = 2 mm) and (−○−, *D* = 3 mm) symbols, for a surfactant concentration of 0.025 mol/l. The dashed line shows the trend of the change of viscosity of water-ethanol mixtures, according to [22] (right axis).

To begin elucidating the underlying physical mechanism of the process, experiments were performed considering mixtures of water and ethanol, varying the volume fraction of ethanol ranging from pure water to pure ethanol, as shown in Fig. 4. It was observed that even when no surfactants are present, the maximum pearl lifetime occurs at approximately 55% of ethanol fraction. Note, however, that the lifetimes for these mixtures are one order of magnitude smaller than those observed for Mezcal. Assuming that the dimensionless bubble lifetime (Eq. 2) is only a function of the Bond number (Eq. 1), we can argue that the lifetime of pearls is directly proportional to the fluid viscosity. While density and surface tension of water-ethanol mixtures have a monotonic behavior with ethanol fraction (from the pure water to the pure ethanol values), viscosity does not: its value increases from that of water, reaching a maximum at around 55% of ethanol, to then decrease to reach the value of pure ethanol^22^. The dotted line in Fig. 4 shows how viscosity varies (plotted on the right scale of the figure) with the volume of ethanol. Therefore, we can argue that both fluid properties and surfactant concentration affect the bubble lifetime. Consequently, it may be inferred that the maximum lifetime observed at the ethanol content at 55% is related to the fact that the liquid viscosity has a maximum value at that concentration.

More experiments were conducted with other Mezcal types, from different regions and agave species. For two cases, the alcohol content was also modified in the same manner as described before. The results are shown in Fig. 3(b), where the bubble lifetime for all liquids is shown as a function of alcohol content. The result is essentially the same, the bubble lifetime shows a clear maximum at around 55% of ethanol volume fraction.

### Numerical Results

From the results shown above, it is clear that the bubble lifetime is both dependent on the fluid properties and the amount of surfactants. However, to elucidate the details of the phenomena, we conducted a numerical study. Details of the numerical scheme can be found in the Methods Section.

First, the residence time of bubbles was computed for water-ethanol mixtures, with the aim to validate the numerical method and to gain some insight into the drainage process. Figure 4 shows the numerically obtained bubble lifetime, considering two bubble sizes which are close to the experimental values. The numerical lifetime was calculated by following the procedure described in the Methods Section. The simulations were obtained for a small amount of surfactant, as it is suspected to be the case for laboratory-clean aqueous mixtures. In very close agreement with the experiments, the bubble lifetime increases as the ethanol volume fraction increases, reaching a maximum for concentrations close to 55% of ethanol, to then decrease for higher concentrations. Given the variability of bubble size in the experiments, the quantitative agreement between experiments and numerical results is remarkable.

**Figure 5:**
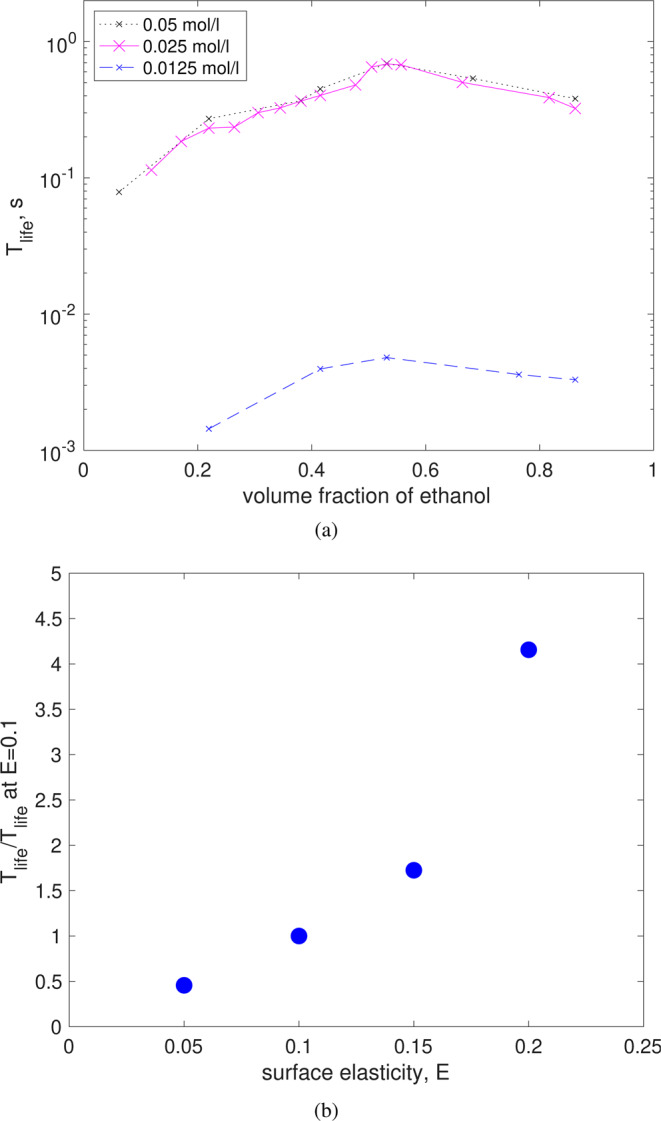
(**a**) Lifetime as a function of ethanol content, for different concentrations of surfactants. (**b**) Maximum lifetime as a function of surface elasticity, *E*; the time is normalized with the case for *E* = 0.1. For all cases, *D* = 2 mm, *c*_0_ = 0.025 mol/l, alcohol content of 55%, Bo = 1.24.

From the experiments, it is clear that the amount and type of surfactants affect the bubble lifetime. We conducted numerical simulations with increasing amounts of surfactants, as shown in Fig. 5(a). The bubble lifetime increases as the ethanol volume fraction increases, reaching a maximum for concentrations close to 55% of ethanol, to then decrease for higher concentrations, in close agreement with the experiments. Most importantly, the maximum lifetime increases with the amount of surfactants; the lifetime increases sharply with the concentration but appears to not increase significantly beyond a certain value of surfactants. These results are also in good qualitative agreement with the experiments. We have conducted many more simulations and analyses to further understand this effect. These results can be found in Atasi *et al.* ^43^.

An interesting and challenging aspect of the Mezcal case is that, in addition to the amount of surfactants, the specific type of it is not known. Furthermore, there may be changes of the surfactant type depending on the species of Agave and production region for each Mezcal. To gain some general understanding in this respect, we also conducted numerical simulations varying the surface elasticity, *E*. This quantity measures the interface resistance to deformation^44^. Fig. 5(b) shows the bubble lifetime as a function of the surface elasticity parameter, considering a fixed value of ethanol content. Clearly, the lifetime increases significantly with the elasticity of the surface. It is important to note that this effect has not been discussed in-depth in the literature. Some initial tests and discussions can be found in Atasi *et al*.^43^.

**Figure 6:**
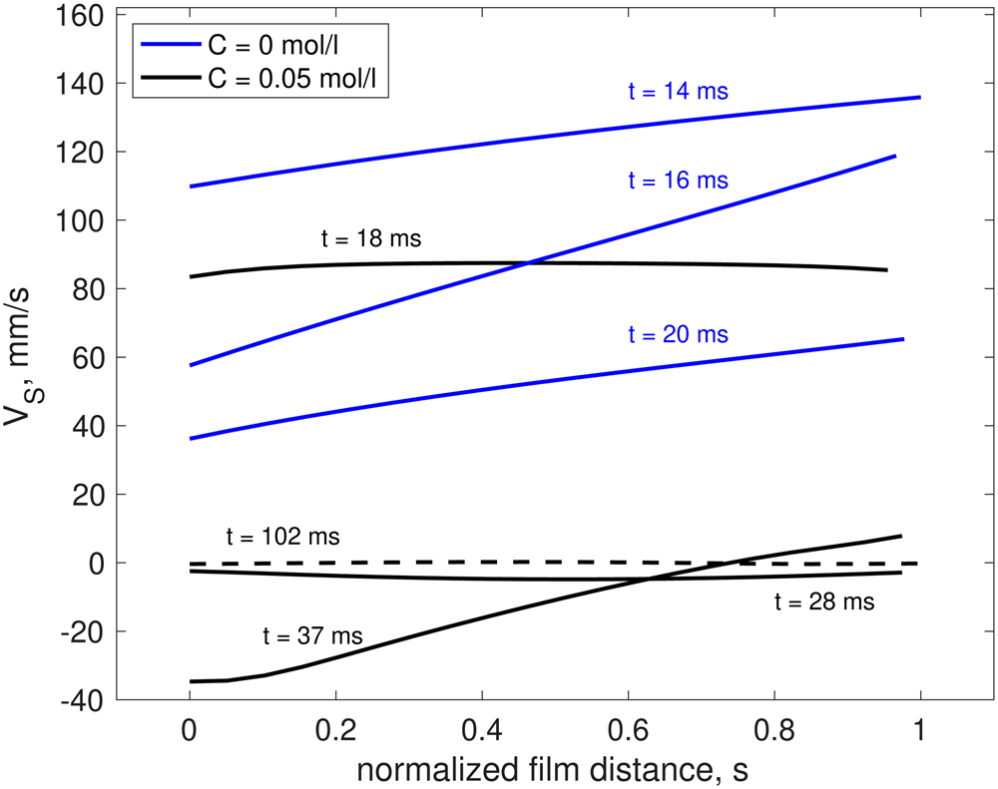
Velocity profile across the film. Tangential fluid velocity, *V*_*s*_, as a function of the normalized distance across the film, *s*, at 0.35 rad from the bubble apex (taking the bubble centroid as reference), for different times and surfactant concentrations. *s* = 0 corresponds to the bubble surface and *s* = 1 refers to the external free surface. The black and blue lines correspond to the cases with and without surfactants, respectively. The dashed line shows the case with surfactacts at a long time.

The numerical simulations can also provide significant insight into the complex drainage process of bubbles in Mezcal. In addition to the effects on the lifetime, details of the flow within the film can also be analyzed. Figure 6 shows the fluid velocity (tangent to the interface) as a function of position within the film, at a certain angle from the apex (*θ* = 0.35 radians) at different times, with and without surfactants. An important difference between the two cases is that speed is significantly larger and uniform (across the thickness) for the case of a bubble without surfactants, in comparison to the case with surfactants. The reason for this reduction stems, first, from the immobilization of the surfaces due to surfactants. Moreover, when surfactants are present, for the angle shown, the fluid at the inner edge of the film (*s* = 0) can move in the opposite direction to that of the gravitational drainage, even for early times (*t* = 37 ms). For later times, *t* = 102 ms the fluid moves at a very small downward speed all across the gap. Note that the simulations consider the effect of surfactant transport but do not contemplate evaporation or temperature gradients. Despite this simplification, the lifetimes obtained numerically are in good agreement with those found experimentally. Therefore, we can argue that surfactant transport dominantly affects the drainage process.

**Figure 7:**
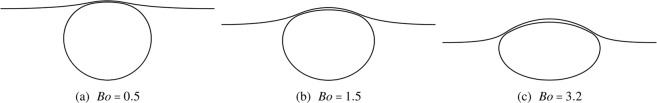
Shape of bubbles obtained numerically for different values of *Bo*.

Additionally, thanks to the unprecedented access to other properties of the flow from the numerical simulations, it is also possible to track the deformability of the interface. We have identified that the film becomes non-uniform along the bubble during the drainage. In particular, a neck region appears for a sufficiently large Bond number, associated to a local thinning of the film as shown in Fig. 7(b,c), which are used to validate the numerical results. Also, the position of the neck changes position from the top of the bubble toward the liquid bath, as the Bond number increases. The appearance of the neck region has been documented for the case of films^38^, but not as extensively for the case of surface bubbles^18^. This thinning zone leads to the rupture of the film which, as shown in Fig. 2(b), generally does not occur at the bubble apex. The results indicate that the position where the rupture starts is also a function of the Bond number.

## Discussion

**Figure 8:**
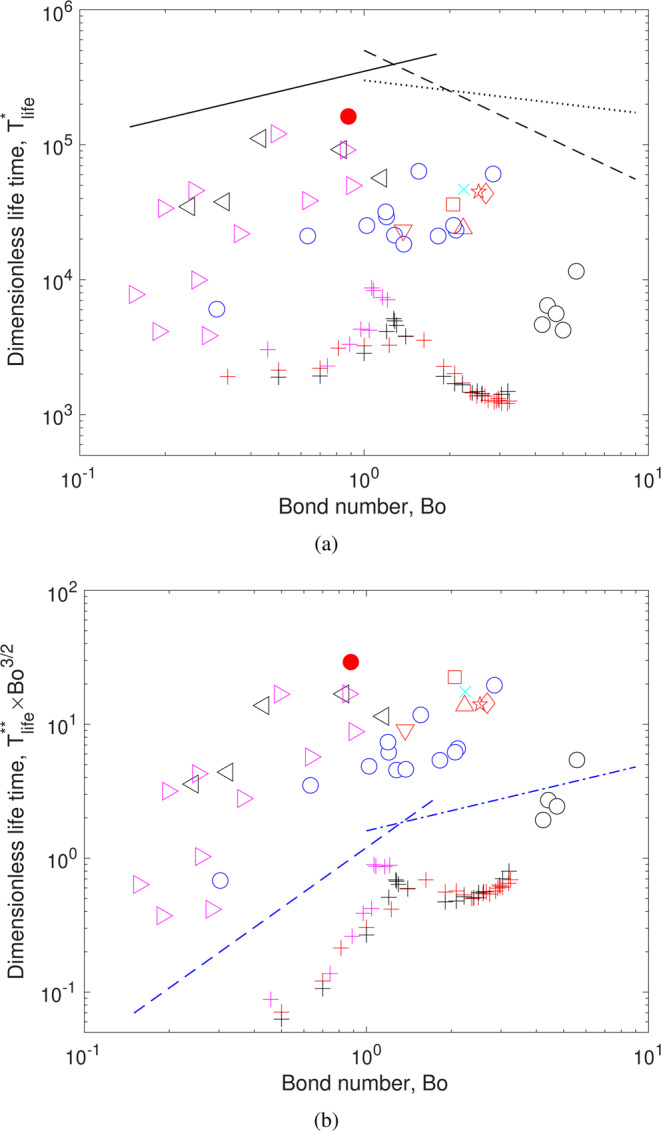
(**a**) Dimensionless lifetime, ${T}_{life}^{\ast }$ as a function of Bond number. The black lines show trends from different predictions: solid line, Eq. (17); dashed line, Eq. (15); and dotted line, Eq. (16). (**b**) Compensated dimensionless lifetime, ${T}_{life}^{\ast \ast }B{o}^{3/2}$ as a function Bond number, *Bo*. Symbols as in Table 1. For clarity, error bars are omitted. The plus symbols (+) are the results of numerical simulations, for a surfactant concentration of 0.025 mol/l, for two different bubble diameters and different ethanol concentrations. The blue dashed and blue dashed-dotted lines show compensated trends predicted by Eq. (13) and Eq. (12), respectively (${T}_{life}^{\ast \ast }B{o}^{3/2}\sim B{o}^{3/2}$ and ${T}_{life}^{\ast \ast }B{o}^{3/2}\sim B{o}^{1/3}$, respectively.).

Let us now recast the bubble lifetimes in dimensionless terms. Considering the scaling from simple inspection (also arising from the extensional flow analysis), the dimensionless lifetime can be calculated according to Eq. (2). Figure 8(a) shows the bubble lifetime, ${T}_{life}^{\ast }$, as a function of the Bond number, *Bo*, defined in Eq. (1). Despite the large dispersion of the data, several key features can be readily identified. First, the dimensionless lifetime of the unaltered Mezcal is higher than any of the other cases, when either water or ethanol was added, or even for other Mezcal samples with other ethanol content. The dimensionless lifetime appears to increase for small values of the Bond number, to reach a maximum at around $Bo\approx 1$, and then decrease as the value of *Bo* continues to increase.

The Bond number allows us to evaluate the driving force for drainage, namely the capillary force for $Bo\ll 1$ and the gravity force for $Bo\gg 1$. The striking feature of the results in Fig. 8(a) is that $Bo \sim 1$ indicates a transition, which coincides with the maximum dimensionless bubble lifetime. The Bond number compares the two main driving forces for drainage: gravitational drainage for large *Bo* and capillary-induced drainage for small *Bo*. Interestingly, the maximum dimensionless lifetime is reached when both effects are of the same order of magnitude.

To further support the existence of a critical *Bo*, we compared our results with predictions from the literature, shown in Fig. 8(a). For *Bo* < 1 the prediction by Howell, Eq. (17), shown by the solid line, shows an increasing lifetime with Bond number. On the other hand, for *Bo* > 1, Eqs. (15) and (16), dashed and dotted line respectively, predict a decreasing trend of the bubble lifetime with *Bo*. These contrasting trend predictions corroborate the existence of a value of *Bo* at which ${T}_{life}^{\ast }$ has a maximum value.

The numerical results, in dimensionless form, are also shown in Fig. 8, along with the experiments. The numerical results show an increasing trend for small *Bo*, but at around $Bo\approx 1$, a clear change of behavior is observed. Note that all simulations, conducted for two different bubble diameters and different ethanol concentrations, collapse into a single band when presented in dimensionless terms. The lifetime of the numerical results is shorter than that in the experiments resulting from the assumptions considered in the simulations. Nevertheless, the qualitative agreement is noteworthy. The cross-over for which gravity and capillary effects are of the same order of magnitude shows a non-monotonous behavior for which a maximum dimensionless lifetime has been observed, both numerically and experimentally. Strikingly, $Bo\approx 1$ corresponds to a bubble size of about 2 mm, i.e. the size for which the lifetime is the longest in the experiments.

One important feature of the dimensionless bubble lifetime, ${T}_{life}^{\ast }$, is its large value, in the order of 10^4^–10^5^ time-scale units. This is an indication that the characteristic time $\mu D/\sigma$ is orders of magnitude smaller than the bubble lifetime. Hence, its magnitude cannot be used to characterize the phenomena properly. Therefore, we use ${T}_{life}^{\ast \ast }$ instead, as defined by Eq. (11). Furthermore, if we consider a compensated dimensionless lifetime, given by the product ${T}_{life}^{\ast \ast }B{o}^{3/2}$, the data dispersion can be reduced by eliminating the dependence on the bubble diameter. Figure 8(b) shows the compensated dimensionless lifetime as a function of the Bond number, *Bo*. The time scaling considers the rupture thickness of the film. As discussed above, the factors that determine the rupture thickness are not completely understood even for pure liquids^18^. In the present case, the presence of particles and surfactants, pose additional difficulties^45^. Hence, since we cannot reasonably assess how the rupture thickness changes for different liquids, we assume that *h*_*rup*_ = 23.5 ± 4.8 *μ*m is the same for all cases. This value corresponds to the measured film thickness for the case of unaltered Mezcal. The data presented in this form shows that the dimensional lifetime increases up to *Bo* of order unity, which is in good agreement with the scaling argument of Eq. (13). For bubbles for $Bo > 1$, the data clearly shows that the compensated dimensionless lifetime is relatively constant. In contrast, the prediction from Eq. (12, shows a slightly increasing trend. Note also that the magnitude of the dimensionless times is of O(10) for all experimental points, which indicates that the characteristic time *t*_*c*_ (Eq. 10) is more representative of the flow phenomena in the draining film. Again, this argument supports the idea that the flow in the film is shear-dominated.

The numerical results are also shown in Fig. 8(b). As in the previous case, the lifetimes obtained from the simulations are shorter for all cases. However, and most importantly, the numerically obtained lifetimes collapse into a single band of data indicating that the scaling is correct. Furthermore, the trend found with the numerical results matches closely with the scaling arguments: the compensated dimensionless time increases for *Bo* < 1 and is roughly constant for *Bo* > 1. One of the advantages of the numerically obtained times is that the data scattering is greatly reduced, which allows us to observe the trend of results more clearly. From these results, it is further demonstrated that there is a critical *Bo* number, around unity, that characterizes a long bubble lifetime.

In summary, we conducted an experimental and numerical investigation to elucidate the phenomena behind the ‘pearls of Mezcal’. By observing the extended lifetime of superficial bubbles, artisanal Mezcal makers have been able to identify the correct amount of ethanol in this distilled spirit without the need for any other measuring device. Intrigued by this empirical technique, we conducted experiments to test the technique under controlled conditions. Single bubbles were generated into a small container, and the time from reaching the surface until bursting spontaneously was measured. It was found that, indeed, bubbles in samples with ethanol volumetric content close to 55% had a characteristically long lifetime. We have found that, in dimensionless terms, the lifetime of a surface bubble increases with Bond number, reaching a maximum value at $Bo\approx 1$, and then decreases for large values of *Bo*. We found that both an increase of the liquid viscosity and the presence of surfactants are needed to observe a long lifetime of bubbles. These factors explain why pearls in Mezcal have a particularly long life at a certain concentration of alcohol and a certain size. Clearly, the artisanal technique is the result of observation, empiricism and tradition. The insight gained in investigating this technique may help elucidate the fundamental mechanisms of bubble formation and stability in many other contexts. For example, the lifetime of surface bubbles could be used as a diagnostic tool to infer the presence of surfactants in a liquid: if the lifetime is larger than that expected for a pure/clean liquid, then the liquid is most likely contaminated. For instance, the bubble nests of some tropical fish have long lifetimes thanks to the presence of bio-surfactants^15^; short-lived nest could indicate environmental contamination or health problems of the fish. Similarly, the contamination levels of water bodies could be correlated to long-lived foam and bubbles, as discussed by Zenit and Rodriguez-Rodriguez ^46^. Furthermore, the lifetime of surface bubbles in applications such as froth floatation or anti-bubble formation could be adjusted by choosing the ‘correct’ surfactant to tune particle or gas capture rate^16,47^. Finally, recent reports indicate that the rupture process of surface bubbles plays a significant role in the spread of infectious diseases^48^; hence, it is important to fully understand the nature of this process. The production of aerosols resulting from bursting bubbles could be reduced if the effects of surfactants are fully understood. The results presented in this investigation may shed some light into such processes of current importance.

## Materials and methods

### Informed consent

The person who appears in Fig. 1(a) and in the S1 Video in the Supplementary Information section has given informed consent to be shown in the paper. He understands that the images may lead to identification.

### Test fluids and physical properties

The physical properties of all the test liquids are reported in Table 1. The origin of the Mezcal samples is also given. According to the Mexican norm, they all come from the Oaxaca state region. Seven Mezcal types were considered; three cases (M1, M6 and M7) were purposely altered to change their water or alcohol content. Also, pure water/ethanol mixtures were tested.

The surface tension was measured with a tensiometer (DynaTester, SITA). The density was measured with a 25 ml pycnometer. The alcohol content was inferred from the density measurement, considering room temperature, *T*_*room*_ = 23 °C. The viscosity was not measured; considering the alcohol content, its value was assumed to correspond to that of a water-ethanol mixture and was obtained from tables^22^.

### Visualization of bubble formation process

The traditional technique to evaluate the alcohol content is shown in S1 Video (Supporting Information) and Fig. 1(a). The *Maestro Mezcalero* issues a stream of fluid from a long reed, with a round opening of about 2 mm in diameter. The jet impinges onto a small gourd cup, of about 10 cm in diameter. As the cup fills up, a pool of fluid is formed of several centimeters in depth. To reproduce the process in a controlled manner, the traditional reed was replaced by a glass pipette of 25 ml, with approximately the same exit diameter as the reed (2 mm). The sample of Mezcal (or other test liquids) of about 20 ml was placed in the pipette and was held fixed in a vertical position with a laboratory holding bracket. Below the exit of the pipette, a 10 × 10 cm^2^ square transparent container was placed, filled with the same liquid to a depth of 2 cm. The process was filmed with a high-speed camera (FASTCAM-APX, Photron) using diffuse back-lighting at 1500 fps.

### Measurement of lifetime

To accurately measure the lifetime of single bubbles, a simple experimental arrangement was used. A short cylindrical glass container of 1.6 cm in diameter and 1.2 cm in height was filled with the test liquid; the rim of the container was roughened to make it slightly hydrophobic. The amount of liquid was slightly larger than the volume of the container, such that a convex meniscus was formed. The arrangement was placed inside a closed container to minimize environmental disturbances during the experiment. At the side of the container, a needle was inserted through the wall via a plastic stopper plug. Bubbles were formed by slowly pushing air through the needle with a syringe. Since the free surface was slightly convex, when a bubble reached the surface it moved to the center of the container where it could be filmed. The bubble traveled approximately 1 cm through the liquid, lasting approximately 0.03 s from its formation until it reached the surface; the bubble moved slightly on the surface quickly reaching a stationary position. The bubble lifetime was measured from the moment when the bubble no longer moved and until it burst.

Experiments were performed with needles of different gauges ranging from 159 *μ*m to 210 *μ*m of internal diameter, to produce bubbles with slightly different diameters. The setup was illuminated from the bottom with a LED light. The bubble diameter was measured from the image obtained from the top; this measurement overestimates the bubble diameter of an equivalent spherical bubble by approximately 13% for the highest Bond numbers^49^. The process of rupture was measured with two synchronized high-speed cameras (Phantom SpeedSense), one aligned from the top and the other from the side. Different recording rates were considered: since the rupture time could take tens of seconds, an ordinary 30 fps was used to determine the bubble lifetime. To measure the rupture speed of the film, the recording rate was as high as 5,000 fps. All experiments were performed under standard laboratory conditions. The container was thoroughly rinsed with distilled water prior to each experiment.

### Thickness of the film during bursting

The thickness of the film can be inferred from the speed at which the leading piercing rim moves as the bubble bursts. Considering the Taylor-Culick velocity^50,51^:

18 $$V=\sqrt{\frac{2\sigma }{\rho {h}_{rup}}}$$

where *σ* and *ρ* are the surface tension and liquid density, respectively. *h*_*rup*_ is the thickness of the film. Experiments were conducted using fluid *M*1 and bubbles with the same diameter (*D* = 1.9 mm).

### Numerical simulations

Direct numerical simulations were conducted by solving the Navier-Stokes equations coupled with the Level-Set method. We refer the reader to Atasi *et al.*^32^ and Abadie *et al.*^52^ for a detailed description of the method and its validation. Briefly, Navier-Stokes equations are solved for two Newtonian and incompressible fluids using the finite volume method (second-order accurate in time and space). Continuity is ensured through a projection method, and the capillary contribution is considered through the classical Continuum Surface Force method. The interface position is tracked using the Level-Set method where the transport of the signed distance to the interface is controlled through the re-distancing techniques. The novel aspect of the numerical scheme presented here is its ability to account for the surfactant concentration both in the liquid and on the interface as given by^53^:

19 $$\frac{\partial C}{\partial t}+{V}_{c}\cdot \nabla C=\nabla \cdot ({D}_{c}\nabla C)$$

20 $$\frac{\partial \varGamma }{\partial t}+{\nabla }_{S}\cdot ({V}_{S}\varGamma )={D}_{s}{\nabla }_{S}^{2}\varGamma +{S}_{\varGamma }$$

where ***C*** is the surfactant concentration in the liquid phase, Γ is the surfactant concentration on the gas-liquid interface, *D*_*c*_ and *D*_*s*_ are the diffusion coefficients of the surfactants in the liquid phase and along the interface, respectively, **V**_*c*_ is the velocity field of the liquid phase, **V**_*S*_ is the projection of **V**_*c*_ on the tangent to the interface, ${\nabla }_{S}=(\bar{I}-n\times n).\nabla$ is the surface gradient operator and *S*_Γ_ is the flux of surfactants from the liquid phase to the interface, due to the adsorption/desorption of the surfactants, i.e., ${S}_{\varGamma }=({D}_{c}{\bf{n}}\mathrm{}.\nabla C){|}_{I}$, where the subscript *I* denotes the bubble-liquid interface. It is given by^54^:

21 $${S}_{\varGamma }={k}_{a}{C}_{I}({\varGamma }_{\infty }-\varGamma )-{k}_{d}\varGamma ,$$

where *k*_*a*_ and *k*_*d*_ are the adsorption and desorption kinetic constants, respectively, and *C*_*I*_ is the surfactant concentration in the liquid in contact with the interface.

It is assumed that the surface tension depends on the surfactant concentration on the interface according to an equation of state derived from the Langmuir adsorption isotherm:

22 $$\sigma ={\sigma }_{0}\left(1+Elog\left(1-\frac{\varGamma }{{\varGamma }_{\infty }}\right)\right)$$

where $E=RT{\varGamma }_{\infty }/{\sigma }_{0}$ is the surface elasticity parameter, *R* is the ideal gas constant, *T* is the absolute temperature, *σ*_0_ is the surface tension of the clean interface and Γ_∞_ is the maximum packing concentration of surfactants on the interface. The surface elasticity, which relates the surfactant concentration on an interface to its surface tension, has a great influence on the drainage of the liquid film atop of a bubble. In particular, for given flow conditions, the elasticity parameter dictates the strength of the Marangoni stress induced by the surfactant concentration gradient on the interface and the consequent immobilization of this interface. Typical values of the elasticity parameter for soluble surfactants in aqueous solutions are *E* = 0.05–0.25^55^.

The numerical solution of these equations is extensively described in Atasi *et al*.^32^. The proper implementation of each term, in particular, the surfactant transport on the gas-liquid interface and the computation of the resulting Marangoni stress, has been verified by adapted validation cases and a free rising bubble situation was compared with results from the literature.

### Mesh

**Figure 9:**
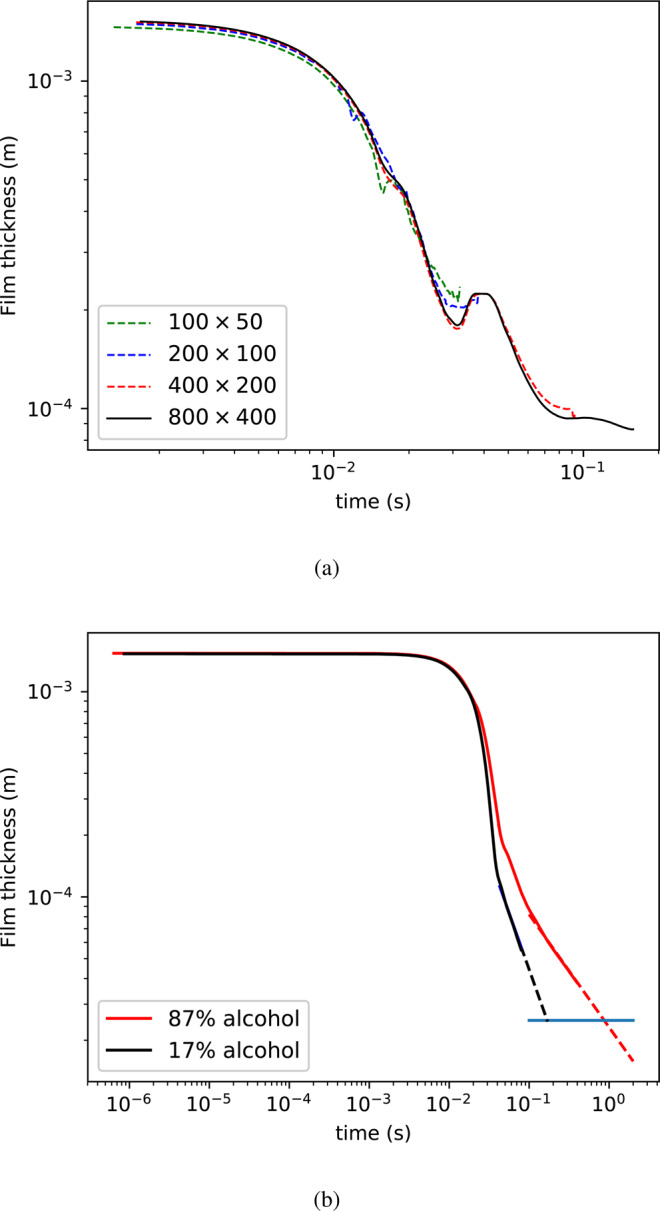
(**a**) Mesh convergence tests. The film thickness is shown as function of time. In all cases, the conditions are the same; only the refinement is varied.; ethanol volume fraction 0.2643, *Bo* = 2.36, surfactant concentration in the liquid bulk *c*_0_ = 0.025 mol/l, surfactant elasticity $E=RT{\varGamma }_{\infty }/{\sigma }_{0}=0.1$. (**b**) Illustration of the procedure to determine the bubble lifetime. In both cases D = 2 mm, *c*_0_ = 0.025 mol/l, *E* = 0.1. The Bond number is 0.87 and 1.35 for the 17% and 87% ethanol content tests, respectively.

Simulations were performed with a non-uniform axisymmetric orthogonal mesh characterized by 400 and 200 cells in the vertical and radial direction, respectively. The mesh size was refined in the vicinity of the interface to be able to properly capture both the film drainage and its rupture. A mesh convergence test was conducted by simulating the same case with increasing number of grid cells, as shown in Fig. 9(a). Clearly, the draining behavior is captured correctly for by the mesh size chosen (400 by 200) and higher.

We have observed that the numerical rupture of the film occurs when the film thickness is about ${h}_{rup}^{(num)}\approx 5\varDelta$ where Δ is the grid size. From the experiments (see above), the film thickness at rupture is *h*_*rup*_ ≈ 24 *μ*m. Due to computing limitations, the minimum grid size we could apply in the numerical simulations was Δ = 10 *μ*m, i.e. ${h}_{rup}^{(num)}\approx 50\mu {\rm{m}}$. For the case of film drainage between two rigid interfaces, and in the limit $Bo\ll 1$, the thinning at the apex follows $h\propto {t}^{-1/2}$ ^35^. Consequently, a decrease by a factor two for the critical film rupture should imply an increase by a factor four of the lifetime. Though this correction factor is certainly a good approximation, it was not applied since the drainage dynamics with surfactants can significantly differ from the one between rigid interfaces, especially at $Bo\approx 1$ and for $Bo\gg 1$.

To reconcile the calculated numerical bubble lifetimes with those measured experimentally, an extrapolation scheme was adopted, shown schematically in Fig. 9(b). Since the evolution of the film thickness showed a power-law dependence, the normalized film thickness was fitted to $h/{h}_{o}=a{t}^{b}$. The numerical lifetime was obtained by calculating the time at which the film reached a value of ${h}_{rup}=24\mu {\rm{m}}$, using the power-law fit.

### Physical properties for surfactant modeling

A certain surfactant is considered in the simulation to represent the effect of the interface contamination. The corresponding properties are ${\varGamma }_{\infty }/{\sigma }_{0}=0.1/(RT)$, where *R* is the ideal gas constant and *T* is the absolute temperature, *k*_*a*_ = 3 m/(mol.s), *k*_*d*_ = 1 s^−1^ and *C*_∞_ = 25 mol/m^3^, *D* = 10^−9^ m^2^/s and *D*_*s*_ = 10^−14^ m^2^/s. The fluid properties correspond to water-alcohol mixtures^22^.

### Validation

The numerical results were extensively validated in Atasi *et al.* ^32^. One additional validation was conducted for this study, considering the shape of bubbles floating on the surface as the film drains. The shape that the bubble adopts while resting on the free surface changes with the value of the Bond number as shown in Fig. 7. The predictions are in close agreement with recent experiments^49^. More importantly, the simulations do capture the retarded drainage due to surface tension gradients induced by concentration gradients of surfactants along the interfaces, as discussed in the paper.

## Supplementary information

Supplementary Information.

Supplementary Information 2.

Supplementary Information 3.

Supplementary Information 4.
