# Supplementary material for: Bubbles determine the amount of alcohol in Mezcal
Source: Sci Rep. 2020 Jul 3;10:11014. doi: 10.1038/s41598-020-67286-x (PMC7335208; doi:10.1038/s41598-020-67286-x)
Supplement: Supplementary file 1 — Supplementary Information. [file 41598_2020_67286_MOESM1_ESM.pdf]

Supplementary Information:
Bubbles determine the amount of alcohol in Mezcal

by G. Rage, O. Atasi, M. M. Wilhelmus, J. F. Hernández-Sánchez, B. Haut, B. Scheid, D. Legendre and R. Zenit

Video S1. Traditional technique. This video shows the traditional ‘pearls of Mezcal’ technique described in the text. The test is conducted by a *maestro mezcalero*, from the Sanzekan Mezcal workshop in Chilapa de Álvarez, Guerrero. The video was taken by L. Diaz-Damacillo in 2016.

Video S2. Bubble formation. The video shows a stream of Mezcal splashing into a container. The video was taken at 1000 frames per second, shown at 30 frames per second.

Video S3. Bubble bursting. The video shows a Mezcal bubble spontaneously bursting 5000 frames per second, shown at 30 frames per second. Both top and side view are shown simultaneously.
